# Nerve transfers improve upper limb function in children with acute flaccid myelitis

**DOI:** 10.1016/j.bas.2025.105876

**Published:** 2025-11-21

**Authors:** Marga Serafimova, Justus L. Groen, Martijn JA. Malessy, Willem Pondaag

**Affiliations:** a Department of Neurosurgery, Leiden NerveCenter, Leiden University Medical Center, the Netherlands; b Department of Neurosurgery, Peripheral Nerve Unit, Tel Aviv Sourasky Medical Center, Israel

**Keywords:** Acute flaccid myelitis, Brachial plexus, Central Nervous System Viral Diseases / surgery∗ [MESH], Nerve Transfer / methods∗[MESH], Child[MESH]

## Abstract

**Objective:**

Acute flaccid myelitis (AFM) is a rare disease affecting the spinal cord resulting in flaccid weakness that may involve any limb and can lead to significant long-term sequalae.

**Methods:**

Collected were results on children affected with AFM who were treated with nerve transfer surgery between 2016 and 2024 in a tertiary referral centre.

**Results:**

Eight children underwent 13 upper limb nerve transfers. Surgery was performed on average 15 months after disease onset. The average follow-up time was 27 months. All but one child showed improvement in upper limb function following surgical intervention. Procedures directed at elbow function deficits had better functional outcome compared to nerve transfers for shoulder function.

**Discussion and conclusion:**

Standard nerve transfers used for brachial plexus injuries were often not possible in these children due to donor nerve deficits. Alternative and less utilized transfers are discussed. We concluded that selected nerve transfers are a reliable method to restore function in children with AFM.

## Highlights

• Acute flaccid myelitis (AFM) is a rare disease affecting peripheral nerve function in children.
• We describe the first report from a European center on 13 nerve transfers for upper limb function restoration in AFM.
• Nerve transfer surgeries contributed to functional improvement of the arm in selected patients.
• Nerve transfers that have become standard for brachial plexus injuries are not always possible in AFM.

## 1 Introduction

Acute flaccid myelitis (AFM) is a rare polio-like disease seen in young children, affecting motor while sparing sensory function. The aetiology is not clearly understood, but it is considered an inflammatory or autoimmune response secondary to a viral infection, commonly Enterovirus D68. Several endemic occurrences world-wide have been described since 2014 involving different viral strains (Vawter-Lee et al., 2021). Since 2014 only 739 cases have been reported in the USA during ten years of registration by the Centre for Disease Control and Prevention (https://www.cdc.gov/acute-flaccid-myelitis/cases-in-us.html). An epidemiological study conducted in the Netherlands found an estimate of 0.06 infected in every 100,000 children per year (Helfferich et al., 2022).

Symptoms of AFM are acute onset of flaccid weakness in one or more limbs with progressive deterioration, frequently following a viral prodrome (Murphy et al., 2021). AFM often leaves the affected child with a chronic neurological deficit, with worse patterns of recovery in proximal muscle groups (Murphy and Pardo, 2020). Full neurological recovery is reported in less than 10 % of cases and can take up to 20 months (Knoester et al., 2019; Martin et al., 2017). Thus far, no predisposing factor has been found to correlate with spontaneous neurological improvement (Andersen et al., 2017).

Due to the rareness of the condition, the heterogenicity of the symptoms and the unravelled pathophysiology of AFM, diagnosis is commonly delayed. MRI of the spine and brainstem show hyperintense grey matter lesions seen on T2 sequencing, without contrast enhancement (Maloney et al., 2015). CSF analysis and nerve conduction studies (NCS) are sometimes used as part of the work up of differential diagnosis (Knoester et al., 2019).

Treatment options are limited in both the acute and in the chronic periods of the disease (Ide et al., 2021; Hopkins, 2017). Steroids, intravenous immunoglobulins and plasmapheresis have been used in the acute phase of neurological symptoms, yet there is no evidence to support their benefit (Dinov and Donowitz, 2022). Currently, no guidelines or expert recommendations exist for the treatment of AFM and management is largely supportive (Hopkins et al., 2021). Surgery for nerve and tendon transfers are options available to improve motor function of the upper extremity.

We present a case series of children affected by AFM treated with nerve transfer surgery. To our knowledge this is the first published data on nerve surgical treatment of AFM originating from a European centre.

## 2 Methods

### 2.1 Patients

Retrospective data was collected on children with Acute Flaccid Myelitis treated at the Leiden NerveCenter between 2016 and 2024. All children underwent nerve surgery to restore shoulder and/or elbow function. Shoulder function was measured by examining shoulder abduction and external rotation and was noted in degrees of active range of motion and/or Mallet subscale (Mallet, 1972). We categorized the result as good/fair/poor. We derived our criteria for the shoulder function from the Mallet classification: for external rotation (with the shoulder in adduction) a good result was considered >20°; for abduction good was >90°, and poor <30°. For elbow flexion and extension, we employed the Medical Research Council (MRC) classification: MRC 4 was considered good, MRC 3 fair and less than MRC 3 was considered poor. Patient demographics including age at diagnosis, gender, and status of Enterovirus D68, as well as details on the surgical procedure, follow-up period, time from diagnosis to treatment, neurological status preoperatively and at last documented follow up were extracted and analysed. In addition, we reviewed the histopathology of nerve samples obtained during the surgery.

Fourteen children with AFM and persistent neurological deficit were examined at the outpatient clinic between 2016 and 2024 and evaluated for possible surgical intervention. Children were selected for nerve transfer if paralysis of relevant muscles did not start to recover six months after onset of AFM. Except for one child, in all cases spontaneous recovery was allowed for at least nine months.

Five children were considered not suitable candidates for nerve transfer surgery. Two children exhibited spontaneous improvement. Two had severe lower limb weakness without available donor nerves for transfer. One child suffered weakness involving all four limbs in addition to left phrenic nerve damage with the need for prolonged ventilation that consequently delayed initial assessment. No suitable nerve donors were considered sufficient for reconstruction for any of the extremities or the phrenic nerve.

### 2.2 Surgical procedure

All surgeries were performed under general anaesthesia without the use of muscle relaxants. Donor and recipient nerves were dissected with standard nerve surgical approaches. Direct selective stimulation was performed with a bipolar forceps connected to a pulse generator (Braun-Aesculap, Tuttlingen, Germany) with increasing voltage (maximum 6V). Recipient nerves were cut only when electrical stimulation did not show any response in the target muscle and an appropriate donor nerve was available. The donor nerve was chosen based on preoperative clinical examination and on strong muscle response upon direct electrical stimulation during surgery. Samples of donor and recipient nerves were sent for histology when surplus length allowed extra trimming of the nerves. No frozen sections were examined; the samples were sent in for the purpose of documentation.

## 3 Results

Demographics of the cohort are presented in Table 1. The average age of onset of AFM was 3.5 years (range 1–5 years). The mean time interval between onset of disease and first evaluation at our clinic was 10 months (range 3–27 months). Children underwent surgery at an average of 15 months (8–29 months) after onset of AFM. The average follow-up time was 27 months (12–50 months).

**Table 1:** Patient demographics, neurological deficit and nerve transfers performed.

No	Age (y)	Gender	D68	TBOC (m)	TBOS (m)	FU (m)	Neuro deficit	Nerve transfers
**1**	4	Male	+	18	21	10	AxN, MCN, Rad	ICN 3 4 5 6 – MCN
**2**	5	Male	N/S	27	29	50	AxN, MCN, Rad, Median, Ulnar	ICN 3 4 5 – MCN
**3**	2	Male	+	3	8	35	AxN, SSN, MCN, Rad Ipsilateral lower limb	XIN – SSN, TDN – AxN, ICN 3 4 – Tri
**4**	5	Female	+	5	11	34	AxN, Rad (triceps) > MCN	TDN – AxN, Rad – Tri
**5**	1	Female	N/S	7	12	23	AxN, Rad Bilateral lower limbs	Tri – AxN
**6**	3	Male	–	8	15	24	AxN, MCN, Rad (triceps) > MCN	Rad – AxN, Uln – Tri
**7**	4	Male	+	5	10	25	AxN, SSN, MCN, Rad	XIN – SSN, Uln – MCN
**8**	4	Male	+	10	13	12	AxN, MCN, Rad	Tri – AxN

No: patient number; Age: age at AFM onset (years); D68: Enterovirus D68 status; TBOC: Time between onset and 1st consultation (months); TBOS: Time between onset to surgery (months); FU: Follow-up (months); Neuro deficit: Neurological deficit at presentation; Procedures: Nerve transfer procedures.

Abbreviations: N/S Not specified, AxN Axillary nerve, MSC Musculocutaneous nerve, Rad Radial nerve, ICN Intercostal nerve, XIN Spinal Accessory nerve, Uln Ulnar nerve fascicle, SSN Suprascapular nerve, TDN Thoracodorsal nerve, Tri triceps branch.

The neurological deficit and the performed nerve transfers are also represented in Table 1. All children had a shoulder abduction deficit, in some external rotation had remained intact. Two children (1 and 2) had severe deficit of hand function. Four children had weakness of the legs in the acute stage, which had recovered fully in two by the time of referral.

Nine children were operated on, eight of them underwent a total of 13 nerve transfers. One child was scheduled for a triceps branch to axillary nerve transfer. During the exploration phase of the surgery, direct electrical stimulation of all triceps branches of the radial nerve did not evoke strong enough muscle responses, making the nerve transfer not feasible. No other regional donor nerves were available, so this surgery was aborted, and no nerve transfer was performed.

Out of the 13 nerve transfers, seven were performed to restore shoulder function (Table 1, Table 2). Two of them were nerve transfers from spinal accessory to suprascapular nerve (children 3 and 7) and five targeting the axillary nerve using the thoracodorsal nerve (children 3 and 4), triceps branches (children 5, 8) and fascicles of the radial nerve proper (child 6) as donors. All but one of the children improved in shoulder abduction by at least 50°; one achieved full active range of motion in the coronal plane after a triceps to axillary nerve transfer (Table 2). One child did not show recovery of abduction at the documented 1-year follow-up; however improvement will hopefully take place with longer follow-up. External rotation of the shoulder improved following all procedures with a minimum of 30°.

**Table 2:** Results of nerve transfers, grouped by recipient nerve.

Function	Recipient Nerve	Donor Nerve	(n)	Result	No	Preoperative exam	Postoperative exam
**Shoulder (ROM)**	SSN	XIN	2	2 good	3	Abd M0 Ex rot M0	Abd 50° Ex rot 55°
					7	Abd M0 Ex rot M0	Abd M0 Ex rot 30°
					3	Abd M0 Ex rot M0	Abd 50° Ex rot 55°
	AxN	TDN	2	2 fair			
					4	Abd M0 Ex rot M2/M3	Abd 60° Ex rot 70°
		Rad∗	3	2 good	5	Abd M0 Ex rot M2	Abduction 100° Ex rot 85°
					6	Abd M0 Ex rot 90°	Abd 180° Ex rot 90°
				1 poor	8	Abd M0	Abd 0°
**Elbow flexion (MRC)**	MCN	ICN	2	2 good	1	M0	M4
					2	M0	M4
		Uln	1	1 good	7	M3	M5-
**Elbow extension (MRC)**	Tri	Rad	1	1 good	4	M0	M4
		Uln	1	1 good	6	M0	M4
		ICN	1	1 fair	3	M0	M3

Shoulder: active range of motion (in degrees) for Abduction (abd) and External rotation (ex rot). External rotation was assessed with arm in adduction with the flexed elbow at the side. (n): number of procedures. No: patient number.

∗ 2 children had triceps branch to axillary nerve transfer, 1 child (child 6) had a transfer of a fascicle from the radial nerve proper.

Abbreviations: M0 – MRC grade 0, SSN Suprascapular nerve, XIN Spinal Accessory nerve, TDN Thoracodorsal nerve, AxN Axillary nerve, MCN Musculocutaneous nerve, ICN Intercostal nerve, Tri Triceps branch, Uln Ulnar nerve, Rad Radial N.

Three nerve transfers for elbow flexion were performed. In two cases (children 1 and 2), intercostal nerves (ICN) were used as donors for the musculocutaneous nerve (MCN). Biceps muscle recovery reached MRC 4 in both children. One child underwent an ulnar fascicle to MCN transfer (child 7). On preoperative exam the elbow flexion was scored MRC 3, however there was no palpable contraction of the biceps muscle or active supination. This was executed by either brachioradialis muscle or wrist extensors (Steindler effect) (Hofstede-Buitenhuis et al., 2011). During surgery the MCN did not respond to electrical stimulation and an ulnar fascicle to MCN transfer was performed, resulting in MRC 5- elbow flexion.

Nerve surgery to restore elbow extension was carried out in three children. All three had strong wrist and finger extension, but weak triceps muscle. In one case a radial nerve fascicle was transferred to a triceps branch (child 4) resulting in MRC 4 in elbow extension. In this child intraoperative electrical stimulation of the radial nerve revealed good motor responses in the brachioradialis muscle, wrist, and finger extensors but not in the triceps muscle. A fascicle of the ulnar nerve was used in another patient (child 6) which resulted in strong elbow extension (MRC 4). The third patient (child 3) with paralysis of the triceps muscle underwent intercostal nerve transfer (intercostals 3 and 4) to the triceps branch and achieved strength of MRC 3.

In six out of the eight patients, the donor and/or recipient nerves involved in the transfers were sent for histopathological examination (Table 3). All examined nerves showed a normal architecture without neuroma formation. Myelination was assessed semi-quantitatively using an osmium staining (Malessy et al., 1999). Eight of the donor nerves were examined and all showed at least 75 % of myelination. Two out of the ten recipient nerves showed 50 % myelination, five showed at least 75 % myelination and two had normal myelination.

**Table 3:** Histopathology of nerve samples sent during nerve transfer surgery.

Patient	Donor		Recipient	
	Nerve	Myelination	Nerve	Myelination
2	ICN	100 %	MCN	>75 %
3	XIN	100 %	SSN	100 %
	TDN	100 %	AxN	100 %
	ICN	N/S	Tri	50 %
4	TDN	75 %	AxN	75 %
	Rad	100 %	Tri	50 %
6	Rad	>75 %	AxN	>75 %
	Uln	>75 %	Tri	>75 %
7	XIN	100 %	SSN	100 %
	Uln	N/S	MCN	100 %
8	Rad	>75 %	AxN	>75 %

Abbreviations: MCN Musculocutaneous nerve, ICN Intercostal nerve, SSN Suprascapular nerve, TDN Thoracodorsal nerve, XIN Spinal Accessory nerve, TDN Thoracodorsal nerve, Rad Radial nerve, Uln Ulnar nerve, Tri Triceps branch, N/S Not specified.

## 4 Discussion

AFM is a serious condition which may cause permanent loss of limb function. We present a retrospective case series of 13 nerve transfers in eight children that improved shoulder and elbow motor function. Several small cohorts (ranging from two to 17 children) report on the nerve surgical strategies to enhance upper limb function in AFM patients that do not recover spontaneously. Additional multiple small cohorts and case reports describe surgical treatment with nerve transfers and external and internal neurolysis (Doi et al., 2019; Patel et al., 2020; Heise et al., 2021; Saltzman et al., 2018; Nath and Somasundaram, 2019; Moore et al., 2021). Current published cohorts originate from South and North America and Asia. While surveillance studies originating from European centres do exist (Harvala et al., 2017), reports on nerve transfer surgery for AFM are lacking.

Our results of nerve transfers in AFM compare well to those previously published in several ways. 1) Recovery of elbow function is better than that of the shoulder. One of the early papers reported 16 children from Shriners Hospital for Children, Philadelphia that underwent various nerve transfer surgeries; the authors concluded that recovery of elbow function was superior to that of the shoulder (Pino et al., 2019). In non-surgically treated children, elbow function also showed better spontaneous improvement as compared to shoulder function. Other series likewise reported that spontaneous recovery occurs in a distal to proximal pattern (Melicosta et al., 2019). 2) Timely nerve transfer results in a better outcome. Some authors suggest nerve reconstruction six to nine months after onset of disease, when clinical improvement does not occur (Rabinovich et al., 2022; Doi et all, 2024). However, this timeframe lacks certainty since spontaneous recovery can occur in a delayed manner. Additionally, surgical treatment was successful in several of our cases despite a longer period of symptoms, which was a result of late referrals. A group from Taiwan reported a cohort comparing surgical to non-surgical treatment (Liao et al., 2007). Their results showed a complete recovery of shoulder abduction in five out of six children that were operated on within one year of illness onset; the child that did not experience significant improvement was operated on three years after AFM onset. Poor results were encountered in their patient series following tendon transfer surgeries, which was hypothesized to result from weakness of the transferred muscles.

Paziuk et al. analysed nerve transfers for shoulder function. Good outcome was described following nerve transfers for the suprascapular nerve (Paziuk et al., 2020). In their series radial to axillary nerve transfer proved to be a reliable donor although details were not provided whether the radial nerve proper or branches to the triceps were used for axillary nerve reinnervation. (Paziuk et al., 2020). In our experience, the triceps branch to axillary nerve transfer was frequently not possible due to triceps weakness. In one case (child 8) a failure occurred (albeit that follow-up was only one year) and another child (case 5) reached abduction of 100° maximum. In one child (case 6) we employed a fascicle of the radial nerve proper as the fascicles to triceps did not respond during intraoperative stimulation, leading to a good result.

Our series confirms that nerve transfer surgery is a useful tool to improve function. Limitations of our study are mainly the retrospective nature of our evaluation and small sample size. As AFM is a very rare disease, even reporting smaller case series may contribute to improving treatment strategies. Data collection was not standardized, although we employed the same outcome measures as we do for brachial plexus birth injury: degrees of active range of movement and Mallet subscales for shoulder movement and MRC grading for elbow movement. This diversity of outcome evaluation together with the heterogeneous nerve transfers did not allow extensive statistical analysis. Additionally, as our centre is a tertiary referral centre for nerve lesions, referral bias most likely has caused a pessimistic outlook on spontaneous recovery.

Little is known about the pathophysiology and histology changes in affected nerves in AFM. Axon loss and demyelination resulting from AFM have been reported in a single case report (Haddad et al., 2021). Here, we included histological evaluation of proximal and distal stumps in several children. We found relatively normal myelination in several recipient nerves, even though these nerves did not exhibit function, neither clinically, nor during intraoperative direct electrical stimulation. In this respect the amount of myelination may not correctly represent axonal loss in AFM. Demyelination was present most markedly in the triceps muscle branches. The diminished myelination is supposedly the result of Wallerian degeneration, congruent with loss of lower motor neurons, located in the anterior horn of the spinal cord. Since sensory axons are not affected in AFM, this may explain the preservation of myelination in the obtained samples. The histological method we used was designed in our hospital to evaluate proximal spinal nerves after traction injury as a way to identify healthy tissue prior to grafting a gap (Malessy et al., 1999), and it is uncertain whether it is also suitable to evaluate changes associated with AFM. In order to understand whether histological abnormalities can interpret nerve transfer success, more comprehensive and larger data are needed.

One of the major challenges of nerve transfers in AFM is the availability of donor nerves. The neurological deficit is asymmetrical and “one of a kind” to each child leading to difficulty in creating a constant treatment protocol. MRI of the spinal cord in the late phase of the disease show focal lesions localized to the anterior horn of the grey matter (Murphy et al., 2021). This radiological pattern is consistent with the clinical “patchy” neurological deficit which does not correspond to specific root levels, or myotomes. The somatotopy of the affected spinal cord segments in AFM does not concur with nerve root levels as in brachial plexus lesions, although data on this subject is scarce. We hypothesize that specific motor neuron pools may be affected in AFM. The anatomy of motor neuron pools in the anterior horn of the grey matter has shown to be organized a) in flexion and extension pools and b) proximal and distal muscles separately (Taitano et al., 2024). Most cases in our study exhibit a pattern of deficit involving proximal more than distal muscles (Table 1), which may fit this known motor neuron pool anatomy. As a result of this specific pattern of neurological deficits, familiar and accepted neurotization strategies for treatment of brachial plexus injury are not always an option in AFM. In brachial plexus injuries, the triceps branches from the radial nerve (C7/C8) can often be used as donors for axillary nerve transfer to restore shoulder abduction (C5/C6). In the current AFM series, the triceps muscle was often paretic, leaving the triceps branches unfit as donors to innervate the axillary nerve. In such cases alternative nerve transfers must be considered. The thoracodorsal nerve was utilized as a donor in two children with reasonable results. Similarly, the ulnar nerve was not always strong enough to be used as a donor in cases of elbow flexion weakness, which made us use the intercostal nerves. For recovery of elbow extension, we used three different donors in three children: intercostal nerves (Goubier et al., 2011, Pino et al., 2019), an ulnar nerve fascicle (Goubier et al., 2019, Pino et al., 2019) and a fascicle of the radial nerve proper distal to its triceps branches. These nerve transfers showed an improvement in triceps force.

Due to the young age of these children, the necessary cooperation during needle EMG was considered insufficient. We selected the donor nerves based on pre-operative neurological assessment and employed intraoperative selective bipolar electrical stimulation to confirm their viability. Even though our pre-operative evaluation did not include nerve conduction studies or needle electromyography (EMG), only one child underwent exploration without the possibility of a nerve transfer.

## 5 Conclusion

The clinical presentation of children with AFM is heterogeneous in terms of rate and extent of spontaneous recovery. Nerve transfers can contribute to functional improvement of the arm in a selected group of children when spontaneous recovery is insufficient. To date, only small series, mainly from the US, have been presented describing the outcomes of nerve transfers. One of the major challenges of nerve transfers in AFM children is the availability of donor nerves. The results achieved with nerve transfer surgery in this European series will contribute to optimizing the indications, timing, and type of nerve reconstructive surgery.

## Informed consent declaration

N/A.

## Disclosures

None.

## Ethical approval declaration

Not required by Dutch legislation for retrospective analysis of case series for patients treated in the authors’ institution.

## Funding

None.

## Declaration of competing interest

The authors declare that they have no known competing financial interests or personal relationships that could have appeared to influence the work reported in this paper.
